# The mechanism and pattern of injuries of undocumented immigrants crossing the Texas-Mexico border along the Rio Grande Valley

**DOI:** 10.1186/s40621-021-00341-x

**Published:** 2021-10-28

**Authors:** Carlos H. Palacio, Bianca Cruz, Cheryl Vanier, Jose Cano, Bradford G. Scott

**Affiliations:** 1 South Texas Health System – McAllen Department of Trauma, 301 West Expressway 83, McAllen, TX USA; 2 Touro University Nevada, Henderson, Nevada USA; 3 Baylor College of Medicine Michael E. DeBakey Department of Surgery, Houston, TX USA

**Keywords:** Undocumented immigrants, Border fence, Injuries, Trauma

## Abstract

**Background:**

Apprehensions of undocumented immigrants in the Rio Grande Valley sector of the U.S.-Mexico border have grown to account for nearly half of all apprehensions at the border. The purpose of this study is to report the prevalence, mechanism, and pattern of traumatic injuries sustained by undocumented immigrants who crossed the U.S.-Mexico border at the Rio Grande Valley sector over a span of 5 years and were treated at a local American College of Surgeons verified Level II trauma center.

**Methods:**

A retrospective chart review was conducted from January 2014 to December 2019. Demographics, comorbidities, injury severity score (ISS), mechanism of injury, anatomical part of the body affected, hospital and ICU length of stay (LOS), and treatment costs were analyzed. Descriptive statistics for demographics, injury location and cause, and temporal trends are reported. The impact of ISS or surgical intervention on hospital LOS was analyzed using an analysis of covariance (ANCOVA).

**Results:**

Of 178 patients, 65.2% were male with an average age of 31 (range 0–67) years old and few comorbidities (88.8%) or social risk factors (86%). Patients most commonly sustained injuries secondary to a border fence-related incident (33.7%), fleeing (22.5%), or motor vehicle accident (16.9%). There were no clear temporal trends in the total number of patients injured, or in causes of injury, between 2014 and 2019. The majority of patients (60.7%) sustained extremity injuries, followed by spine injuries (20.2%). Border fence-related incidents and fleeing increased risk of extremity injuries (Odds ratio (OR) > 3; *p* < 0.005), whereas motor vehicle accidents increased risk of head and chest injuries (OR > 4; *p* < 0.004). Extremity injuries increased the odds (OR: 9.4, *p* < 0.001) that surgery would be required. Surgical intervention was common (64%), and the median LOS of patients who underwent surgery was 3 days more than those who did not (*p* < 0.001).

**Conclusion:**

In addition to border fence related injuries, undocumented immigrants also sustained injuries while fleeing and in motor vehicle accidents, among others. Extremity injuries, which were more likely with border fence-related incidents, were the most common type. This type of injury often requires surgical intervention and, therefore, a longer hospital stay for severe injuries.

## Introduction

The United States (U.S.)-Mexico border extends 1980 miles from San Diego, California to Brownsville, Texas. Hundreds of thousands of undocumented immigrants enter the U.S. illegally every year, and many are apprehended. The number of apprehensions has long been considered a reasonable proxy for the number of illegal border crossings, but recent analyses have suggested that apprehensions are generated by a complex combination of enforcement intensity, number of undocumented immigrants, and migrant behavior (e.g., seeking out border enforcement officers to seek asylum). The number of apprehensions at the southern U.S. border peaked in the 1990s, with an estimated average of 1.3 million people apprehended per year. There were no clear directional trends reported in the number of apprehensions from 2014 to 2019 (Rio Grande Valley Sector, 2020; U.S. Border Patrol Monthly Apprehensions (FY 2000 – FY, 2019).

The geographical patterns of migration apprehensions have been shifting away from borders near the cities of San Diego, California, and Tucson, Arizona, toward a more rural area called the Rio Grande Valley (Texas) Sector. The Rio Grande Valley Sector encompasses 34 counties in Southeast Texas (Rio Grande Valley Sector, 2020). The Rio Grande Valley is a region in South Texas comprised of four counties (Hidalgo, Starr, Cameron and Willacy) along the U.S-Mexico border with an estimated population of 1.4 million people (Quick Facts United States, 2020). Apprehensions in the Rio Grande Valley increased from 13% (59,766 out of 447,731) of total apprehensions in 2010 to a high of 53% (256,393 out of 479,371) of total apprehensions along the southwestern border in 2014 (U.S. Border Patrol Monthly Apprehensions (FY 2000 – FY, 2019; U.S. Border Patrol Southwest Border Apprehensions by Sector Fiscal Year 2020, 2020). During their journey, undocumented immigrants endure countless hardships, sometimes leading to death. It has been estimated that more than 1600 undocumented immigrants died attempting to cross the U.S. Mexico border between 1993 and 1997 (Southwest Border Deaths by Fiscal Year, 2020). The presence of physical barriers and decisions made regarding enforcement policies impact not only deaths, but also the likelihood and types of injuries that occur in this population. There are many potential causes for traumatic injuries in the border region, including rough terrain, assault from humans or wild animals, a water crossing or bridge, and the border fence. The hazards associated with a crossing are specific to the locality, which impacts the types of injuries sustained.

Previous efforts to document the types of injuries sustained by undocumented immigrants crossing the U.S. Mexico border have focused on specific groups crossing the border in areas near large cities. A study of trends in the San Diego, California, area of the border from 2000 to 2007 (Kelada et al., 2010) limited inclusion to patients who had sustained their injury “in an attempt to cross the border on foot by scaling the fence.” Another study, which was focused on medical issues near the border proximate to Tucson, Arizona, reported that the second most common problem was dehydration or heat-related issues (Koleski et al., 2019). There has been no similar study in the Rio Grande Valley, where the relative share of apprehensions has rapidly grown to approach 50% of all apprehensions in the past decade. The fence located along the Southwestern Border in the Rio Grande Valley is a steel barrier 7 m tall, cemented into a 1-m-wide trench (Ramey et al., 2019). Once undocumented immigrants cross this barrier, they risk apprehension by U.S. Customs and Border Patrol (U.S. CBP) agents for illegal entry. A study of the patterns and causes of traumatic injuries in the Rio Grande Valley is needed to inform medical planning and future actions related to physical structures and enforcement.

The purpose of this retrospective chart review was to examine the mechanism and pattern of traumatic injuries sustained by undocumented immigrants while crossing the U.S.-Mexico border in the Rio Grande Valley region between 2014 and 2019. The study took place at an American College of Surgeons (ACS) verified Level II trauma center located approximately 15 miles from the Texas-Mexico border in the Rio Grande Valley Sector. The proximity to the U.S. Mexico border and Level II trauma center status is the primary reason undocumented immigrants who suffer traumatic injuries are transported to the trauma center involved in this study. There are two ACS verified Level II trauma centers located in the Rio Grande Valley region, and the nearest Level I trauma center is over 300 km away. Previous literature related to this topic have come from urban Level I trauma centers that are academic institutions in California, Arizona, and West Texas (Kelada et al., 2010; Koleski et al., 2019; Ramey et al., 2019; Mclean & Tyroch, 2012).

## Methods

### Participants

The trauma registry was queried for patients who arrived between January 2014 and December 2019, were brought by U.S. CBP agents, and who lacked a social security number. After an electronic search to narrow the field based on insurance status (“other,” “government subsidized program”, or “self-pay”), each electronic medical record was manually accessed to verify that patients met inclusion criteria. The Institutional Review Board at the University of Texas- Rio Grande Valley approved this retrospective study even though it was considered exempt and waived the requirement for patient informed consent.

### Data collection

Demographics, comorbidities, social risk factors, country of origin, Glasgow Coma Scale (GCS), injury severity score (ISS), ICU and hospital length of stay (LOS) were obtained from each chart. The mechanism of injury was noted and the anatomical part(s) of the body was categorized as: intracranial, head, face, chest, abdomen and extremities. Extremity injuries were sub-classified as upper or lower and the specific bone(s) involved were documented: humerus, radius, ulna, femur, tibia, fibula, calcaneus, and ankle. Spine injuries were subdivided by region as follows: cervical (C1- C7), thoracic (T1-T10), and thoraco-lumbar spine (T11-L5). Patients with acute spinal cord (ASC) injuries, were classified according to the American Spinal Injury Association (ASIA) score (American Spinal Injury Association (ASIA) Impartment scale, 2020). Surgical intervention was documented and treatment costs were provided by the institution.

### Analysis

Descriptive statistics (number and percent) for demographics, injury location and cause, and temporal trends are reported with no supporting inferential statistical tests. The injury severity scores (ISS) are described based on median and interquartile range due to a highly skewed distribution. Several associations were tested using a Fisher’s exact test: between causes of injury and injury locations, surgery and injury location, and surgery and use of the ICU (coded as yes/no).

The impact of injury severity score (ISS) or surgical intervention on days in the hospital were analyzed using an analysis of covariance (ANCOVA) model with surgery, ISS, and their interaction as predictor variables. Days in hospital and ISS were both log-transformed for the analysis, and descriptive statistics were back-transformed for presentation in tables or figures. For patients who stayed at least 1 day in the ICU, and length of stay in the ICU between those who did and did not have surgery was tested using a Mann-Whitney test. Since 93.3% of patients arrived with a GCS of 15, a formal analysis was not possible, so descriptive statistics were used to qualitatively examine the relationship between GCS and length of stay in the hospital or ICU. Statistical significance was assessed at alpha = 0.01. Analyses were done in Rv3.6.3 (R Core Team. 2020. R: A language and environment for statistical computing. R Foundation for Statistical Computing, Vienna, Austria. URL https://www.R-project.org/).

## Results

The 178 patients included in the study ranged in age from 0 to 67 years and the majority were male (Table 1). Over 97% were of Hispanic or Latino ethnicity, and the country of origin for 89.9% was either El Salvador, Guatemala, Honduras, or Mexico (Table 1). Slightly more than 10% of patients had comorbidities, with hypertension alone, or in combination with other comorbidities, being the most common (8 patients, 4.4%). Self-reported social risk factors were also rare. Tobacco use was the most frequently reported substance used, either alone or in combination with another social risk factor, by 22 patients (12.3%; Table 1). There were no clear temporal trends in the total number of injured patients (2014: 34; 2015: 15; 2016: 19; 2017: 37; 2018: 24; 2019: 49).

**Table 1:** Demographic information, comorbidities, social risk factors for patients included in the study, shown as number of people (percent). Countries of origin with three or fewer patients included Belize, Brazil, China, Colombia, Cuba, Ecuador, Romania, and Venezuela. IQR = inter-quartile range

Number of Patients	178	
**Age**	Median = 30.5	IQR = 23, 38
Under 18	9 (5.1%)	
**Gender**	Male = 116 (65.2%)	Female = 62 (34.8%)
**Country of Origin**	El Salvador	27 (15.2%)
	Guatemala	36 (20.2%)
	Honduras	34 (19.1%)
	Mexico	63 (35.4%)
	Unknown	6 (3.4%)
	Other (< 3 per country)	12 (6.7%)
**Comorbidities**	No Cormorbidities^a^	158 (88.8%)
	Hypertension (HTN)	5 (2.8%)
	Gastroesophageal Reflux Disease (GERD)^b^	4 (2.2%)
	Tuberculosis	3 (1.7%)
	Diabetes Mellitus (DM)	2 (1.1%)
	Asthma	1 (0.6%)
	Hyperlipidemia (HLD)	1 (0.6%)
	HTN&DM	2 (1.1%)
	DM&HLD	1 (0.6%)
	HTN&Cerebrovascular Accident	1 (0.6%)
**Self-reported Social Risk Factors**	No Self-Reported Social Risk Factors^c^	153 (86%)
	Alcohol	2 (1.1%)
	Tobacco^d^	18 (10.1%)
	Alcohol & Tobacco	3 (1.7%)
	Pregnancy	1 (0.6%)
	Intravenous Drug Abuse (IVDA)	1 (0.6%)
	Alcohol & Tobacco & IVDA	1 (0.6%)

^a^includes 8 who did not receive a TB test

^b^includes 1 who did not receive TB test

^c^includes 29 who did not receive a pregnancy test

^d^includes 8 who did not receive a pregnancy test

### Location of injury and ISS

Extremities were the most common injury location (*n* = 108). The majority of these extremity injuries were closed fractures (*n* = 87, 81%). The lower extremities were most frequently injured (*n* = 81, 75%), with tibia (*n* = 42, 51%) and fibula (*n* = 33, 40%) being the most common. The spine was the second most frequently injured body part (Table 2), with compression fractures (*n* = 21 of 36, 58%) and burst fractures (*n* = 6 of 36, 17%) most commonly noted. When subdivided by region, injury to the thoraco-lumbar spine (*n* = 28 of 36, 78%) was most common, followed by thoracic (*n* = 8 of 36, 22%) and cervical (*n* = 3 of 36, 8%) regions of the spine. ASC injury was noted in three patients, two with ASIA A and one with ASIA C. The fourth most frequently injured body part was the chest (Table 2), with the majority of patients presenting with lung contusions (*n* = 12 of 20, 60%) and/or pneumothorax (*n* = 10 of 20, 50%). The fifth and sixth most frequently injured locations were the skull and intracranial region (Table 2).

**Table 2:** Number of patients (% of 178) and injury severity score (ISS), hospital length of stay (LOS), and ICU LOS, reported as median and interquartile range by locations and cause of injury. The number (%) of patients who required intensive care unit (ICU) care (‘Required ICU’) or surgery is also reported

	Number (%)	ISS	LOS Hospital	Required ICU	LOS ICU	Required Surgery
**Injury Location**						
Extremity	108 (60.7)	4 (4,9)	5 (3,7)	18 (16.7)	5 (2,7)	90 (83.3)
Spine	36 (20.2)	10 (8,13)	6 (3,7)	23 (63.9)	3 (2,5)	19 (52.8)
Soft tissue head	26 (14.6)	6 (4,16)	4 (2,7)	14 (53.8)	2 (2,6)	14 (53.8)
Chest	20 (11.2)	9 (5,18)	5 (3,6)	14 (70.0)	3 (2,7)	7 (35.0)
Skull injury	19 (10.7)	17 (7,27)	6 (3,12)	13 (68.4)	7 (2,12)	12 (63.2)
Intracranial	18 (10.1)	19 (9,32)	8 (2,19)	15 (83.3)	7 (2,13)	10 (55.6)
Pelvis/Abdomen	14 (7.9)	17 (8,17)	5 (3,11)	8 (57.1)	7 (3,12)	8 (57.1)
Pelvis	12 (6.7)	15 (7,17)	5 (2,10)	6 (50.0)	7 (4,13)	6 (50.0)
Abdomen	3 (1.7)	21 (19,35)	23 (14,34)	3 (100.0)	11 (6,13)	3 (100.0)
**Injury Cause**						
Assault	18 (10.1)	5 (3,15)	4 (2,5)	10 (55.6)	3 (2,4)	3 (16.7)
Bridge	17 (9.6)	9 (4,12)	6 (5,7)	5 (29.4)	5 (2,5)	14 (82.4)
Fence- jump or fall	60 (33.7)	4 (4,9)	5 (3,7)	15 (25.0)	3 (2,6)	46 (76.7)
Injured fleeing	40 (22.5)	4 (4,9)	4 (3,5)	6 (15.0)	5 (3,5)	29 (72.5)
Motor vehicle accident	30 (16.9)	9 (5,16)	3 (2,8)	19 (63.3)	2 (2,6)	15 (50.0)
Other	13 (7.3)	4 (1,9)	3 (3,5)	3 (23.1)	7 (5,7)	7 (53.8)

**Fig. 1:**
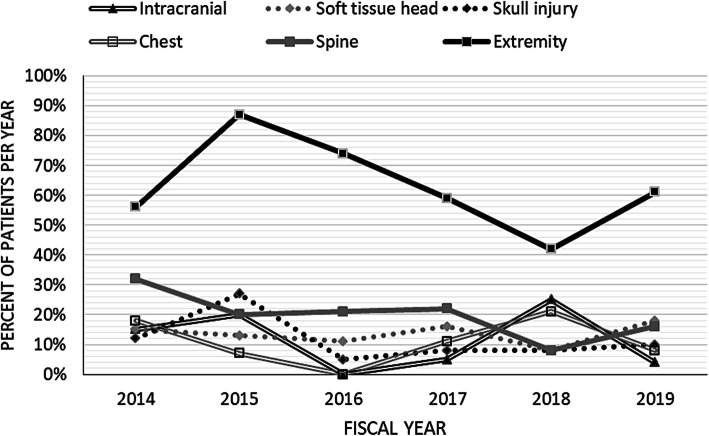
Temporal trends in location of injury. Total number of patients injured is in Fig. 2, and the percent of patients with a particular injury is reported by year. Within-year percentages sum to more than 100% because some patients had injuries in multiple locations

Although variation in injury location was observed over time, extremity injuries were consistently the most common every year of the study (Fig. 1). There were no clear temporal trends in location of injury (Fig. 1). Injury severity score (ISS) was highest in intracranial, skull, pelvis or abdomen injuries and lowest for extremity and soft tissue head injuries (Table 2).

### Cause of injury

**Fig. 2:**
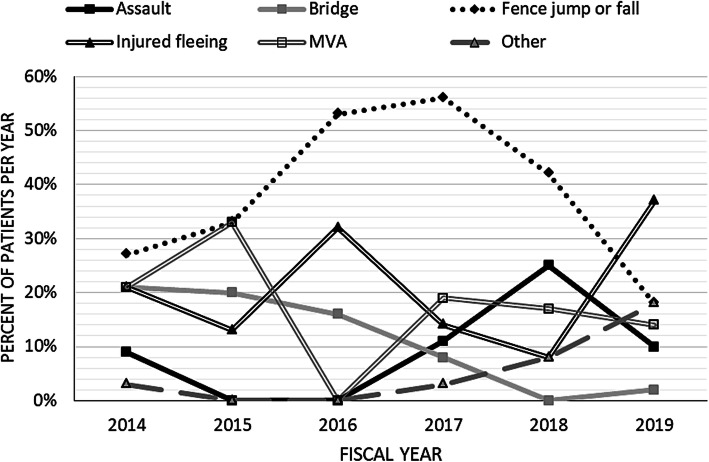
Percent of injuries due to various causes per year. The total number of patients per year: 2014: 34; 2015: 15; 2016: 19; 2017: 37; 2018: 24; 2019: 49

The most common cause of injury was border fence-related (jumps and falls), followed by injury while fleeing and motor vehicle accidents (Table 2). The ISS of patients who were injured on the bridge and in motor vehicle accidents were highest. The lowest ISS was associated with injuries incurred while fleeing or jumping or falling from the border fence (Table 2). The cause of injury was highly variable from year to year. There was a general upward trend in assaults from 2016 to 2019, and injuries associated with the bridge decreased between 2014 and 2018 (Fig. 2).

Some causes of injury were strongly associated with the injury location (Table 3). Border fence-related injuries were associated with a 3.36-fold increase in the odds of an injury to an extremity (Table 3). Patients injured in motor vehicle accidents and assaults were less likely than patients injured by other mechanisms to sustain injuries to extremities. There were no causes of injury that were negatively or positively associated with injuries to the spine, pelvis, or abdomen. Patients involved in motor vehicle accidents had 4- to 8- fold higher likelihood of having soft tissue head, skull, intracranial, or chest injuries. Patients who had been injured in assaults had 6-fold increased odds of having an intracranial injury, though small sample size created high uncertainty in this case (99% confidence interval 1.05, 30.80; Table 3).

**Table 3:** Univariate associations between injury location and injury cause

Injury Location	Injury Cause	Odds ratio	99% CI		***P***-value
Extremity	Assault	**0.16**	**0.02**	**0.72**	**0.001**
	Bridge	1.21	0.28	6.20	0.799
	Fence- jump or fall	3.36	1.29	9.62	0.001
	Injured fleeing	**3.24**	**1.06**	**12.05**	**0.005**
	MVA	**0.18**	**0.05**	**0.58**	**< 0.001**
Spine	Assault	0.21	0.00	2.09	0.128
	Bridge	2.37	0.45	10.59	0.117
	Fence- jump or fall	2.05	0.71	5.87	0.075
	Injured fleeing	0.26	0.03	1.21	0.025
	MVA	1.91	0.51	6.47	0.210
Soft tissue head	Assault	3.47	0.64	15.81	0.029
	Bridge	0.76	0.03	5.15	1.000
	Fence- jump or fall	0.06	0.00	0.58	0.000
	Injured fleeing	0.59	0.08	2.53	0.451
	MVA	**4.08**	**1.06**	**14.95**	**0.004**
Chest	Assault	2.55	0.33	13.41	0.125
	Bridge	1.06	0.05	7.44	1.000
	Fence- jump or fall	0.09	0.00	0.84	0.002
	Injured fleeing	0.16	0.00	1.56	0.049
	MVA	**8.77**	**2.12**	**38.61**	**< 0.001**
Skull injury	Assault	3.96	0.62	20.10	0.028
	Bridge	1.13	0.05	8.01	1.000
	Fence- jump or fall	0.21	0.01	1.28	0.023
	Injured fleeing	**0.00**	**0.00**	**1.04**	**0.008**
	MVA	**7.58**	**1.77**	**33.81**	**< 0.001**
Intracranial	Assault	**6.06**	**1.05**	**30.80**	**0.004**
	Bridge	1.21	0.05	8.65	0.684
	Fence- jump or fall	0.22	0.01	1.39	0.036
	Injured fleeing	0.00	0.00	1.11	0.014
	MVA	**4.95**	**1.07**	**21.94**	**0.003**
Pelvis or Abdomen	Assault	0.67	0.00	7.47	1.000
	Bridge	1.65	0.07	12.58	0.627
	Fence- jump or fall	1.10	0.18	5.46	1.000
	Injured fleeing	0.55	0.02	3.80	0.739
	MVA	1.38	0.12	8.15	0.708

Odds ratio and associated 99% confidence interval (CI) are reported, in addition to the *p*-value from the Fisher’s exact test. Odds ratios with *P* < 0.01 are in bold. *MVA* Motor vehicle accidents

### Surgery and length of stay (LOS)

The majority of patients who were brought to the hospital required surgery every year except 2018 (2014: 79%; 2015: 93%; 2016: 89%; 2017: 57%; 2018: 33%; 2019: 55%; 2014–2019: 64%). Given the high incidence of extremity injuries, it is not surprising that injuries to extremities increased the odds that surgery would be needed by 9-fold (Table 4). Injuries to the chest decreased the odds of needing surgery.

**Table 4:** Univariate associations between injury location and surgery

Injury Location	Odds ratio	99% CI		***P***-value
Extremity	**9.43**	**3.64**	**26.34**	**< 0.001**
Spine	0.55	0.20	1.59	0.124
Soft tissue head	0.61	0.18	2.05	0.272
Chest	**0.26**	**0.06**	**1.00**	**0.006**
Skull injury	0.96	0.24	4.36	1.000
Intracranial	0.67	0.16	2.93	0.446
Pelvis or Abdomen	0.73	0.15	4.01	0.574

Odds ratio and associated 99% confidence interval (CI) are reported, in addition to the *p*-value from the Fisher’s exact test. Odds ratios with *P* < 0.01 are in bold

**Fig. 3:**
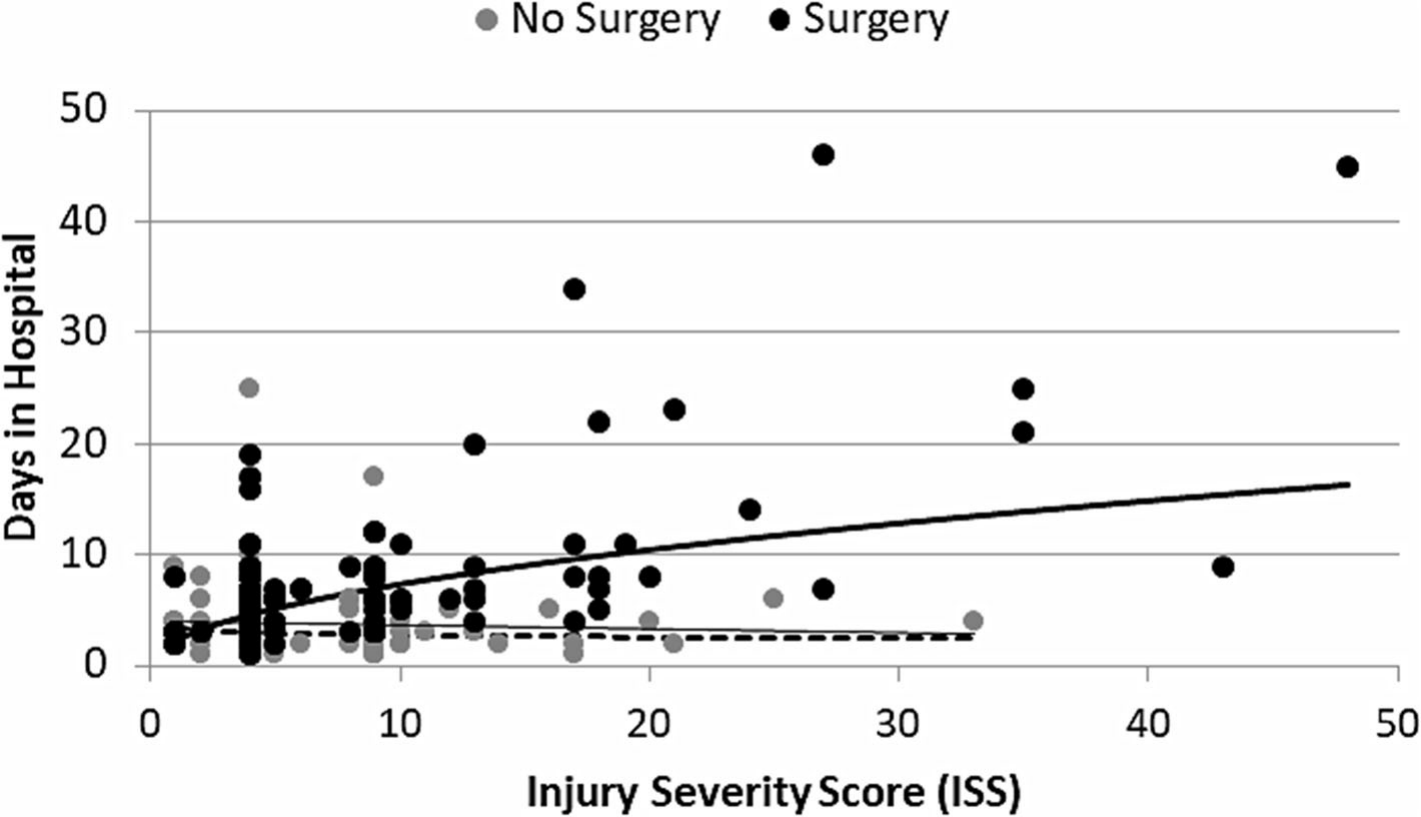
Relationship between ISS and days in hospital (LOS), shown separately for patients who did (solid line) and did not (dashed line) have surgery. Data are shown on linear scale; the regression model was fit with both variables log-transformed to meet assumptions of the models, as reflected in the lines and the equations: Surgery: log10(LOS) = 0.36 + 0.51*log10(ISS); No surgery: log10(LOS) = − 0.09 + 0.53*log10(ISS)

Median length of stay in the hospital was 3 days (inter-quartile range (IQR): 2,4) for patients who did not have surgery, which was shorter than for patients who had surgery (median = 6; IQR = 4,8; W = 1643.5,*P* < 0.001). Both the ISS and the GCS had low to moderate effects on LOS in the hospital or the ICU. The ISS did not impact LOS in the hospital for patients who did not undergo surgery (t = − 0.9, df = 62, *P* = 0.388; R^2^ = 0.01). However, higher ISS predicted longer hospital stays in patients who required surgery (t = 7.5, df = 112, *P* < 0.001; R^2^ = 0.34; ANCOVA interaction term: chi-square = 26.1, df = 1, *P* < 0.001; Fig. 3). The four patients with the longest hospital stays also had the lowest GCS United States, 2020 on arrival (data not shown), but some patients with GCS of 15 also had longer stays in the hospital or the ICU (data not shown). It appears that GCS is one of several contributing factors to determining length of stay.

Approximately one-third (58 of 178) of patients spent time in the ICU. Patients who had surgery were less likely to spend time in the ICU than patients who did not have surgery (no surgery: 45.3% (29 of 64); surgery: 25.4% (29 of 114); chi-squared = 6.5, df = 1, *P* = 0.011). For patients who were sent to the ICU, patients who had surgery spent a median of 7 days in the ICU, whereas patients who did not have surgery spent only 2 (W = 204.5, *n* = 29 for each group, *P* < 0.001).

The majority of patients (*n* = 130, 73%) were discharged back into U.S. CBP custody, followed by being discharged home (*n* = 36, 20%), and 6% (*n* = 10) were transferred to another institution for a higher level of care. The total estimated institutional cost of care was available for 121 patients and was 1.1 million USD.

## Discussion

To the best of our knowledge, this is the first retrospective chart review at a trauma center in the Rio Grande Valley region that describes the various trauma mechanisms and injuries sustained by undocumented immigrants crossing the U.S.-Mexico border. Border fence-related injuries were the most common, like previous studies (Ramey et al., 2019). On the other hand, it has not been previously reported that injuries while fleeing from U.S. CBP agents were also a common mechanism, along with MVAs. Interestingly, traumatic injuries incurred while fleeing from U.S. CBP surpassed border fence-related injuries in 2019. This suggests undocumented immigrants cross natural and/or manmade barriers before being apprehended. Similar to prior studies, young adult males from Latin American countries made up the majority of study population (Ramey et al., 2019; Mclean & Tyroch, 2012).

Our results are similar to prior studies that reported lower extremity injuries are the most common type of injury (Kelada et al., 2010; Mclean & Tyroch, 2012; Burk et al., 2017). Musculoskeletal was also the most common type of injury among undocumented immigrants upon arrival to the Level I trauma center in Arizona (Burk et al., 2017). Furthermore, Kelada et al. found that 83% of patients who fell from the border fence arrived with extremity injuries (Kelada et al., 2010). In this study, 70% of patients whose mechanism of injury was border fence-related presented with a lower extremity injury. Mclean et al. observed comparable rates, with 71% of patients presenting with lower extremity injuries after falls from border bridges. Height, stopping distance and position at impact play an important role on vertical deceleration injuries (Mclean & Tyroch, 2012). However, our study is different in documenting that extremity injuries are positively associated with fleeing, in addition to border fence or bridge related.

Neurologic trauma within this population was the second most common type of injury. Spine injuries were observed more than intracranial injuries. Compression and burst fractures caused by high fall injuries, particularly when landing feet first, have been previously described in this patient population (Ramey et al., 2019; Mclean & Tyroch, 2012). We noticed similar rates, with the thoraco-lumbar spine being the most commonly injured region. Some spinal injuries were quite serious, requiring surgery and ICU admission more than half the time. Unfortunately, two patients suffered complete spinal cord transection. Similar issues have been reported in other articles (Ramey et al., 2019; Mclean & Tyroch, 2012).

Intracranial injuries occurred less frequently than spine injuries. However, they were typically more severe overall and resulted in an increased hospital LOS. Our findings align with Ramey et al., likely due to the severity of the injury and the rehabilitation time necessary to ensure a safe discharge (Ramey et al., 2019).

The ISS score effectively predicted hospital LOS for patients who had surgery. Another factor that contributes to a prolonged LOS is the final disposition of the patient once acute care needs have been met. The value of social workers and case managers in facilitating final disposition cannot be overstated. They collaborate with foreign consulates to locate patient families and help coordinate international transportation for patients transferred back to their country of origin. Caring for the most severely injured patient also translates to an increase in cost of care, oftentimes absorbed by institutions.

Although this study included a small number of patients, treatment costs per patient can be substantial. The total cost of care is difficult to calculate due to the involvement of different services (e.g., laboratory, emergency medicine physician, trauma surgeons, and anesthesiologist) that bill independently of the hospital. The undocumented immigrants who received care at our institution had an immigration health insurance designation documented within their electronic medical record and charges were submitted to the U.S. CBP local office. Treatment cost data for the institution was only available for 121 patients and was 1.1 million USD with a 7.7% reimbursement rate. Our treatment cost data underestimates the total cost of care for this patient population. It is likely that trauma centers along the U.S – Mexico border incur financial losses when providing care for this patient population.

Limitations of this study include its retrospective nature, small sample size, the possibility of missed patients and inability to follow up. Furthermore, a complete cost analysis was not conducted due to incomplete data. Future studies will be aimed at assessing injury prevention strategies for these patient population.

## Conclusion

Undocumented immigrants crossing the U.S.-Mexico border at the Rio Grande Valley are most commonly injured when jumping from or climbing the border fence, fleeing, or riding in high-speed motor vehicles. Extremity injuries, frequently border fence-related, are most common and often require surgical intervention. Surgical intervention contributes to a prolonged hospital LOS, especially for more severe injuries.

## Data Availability

We have that data.
